# Supplementary figures and images for: Prediction of Nodal Metastasis in Lung Cancer Using Deep Learning of Endobronchial Ultrasound Images
Source: Cancers (Basel). 2022 Jul 8;14(14):3334. doi: 10.3390/cancers14143334 (PMC9321716; doi:10.3390/cancers14143334)

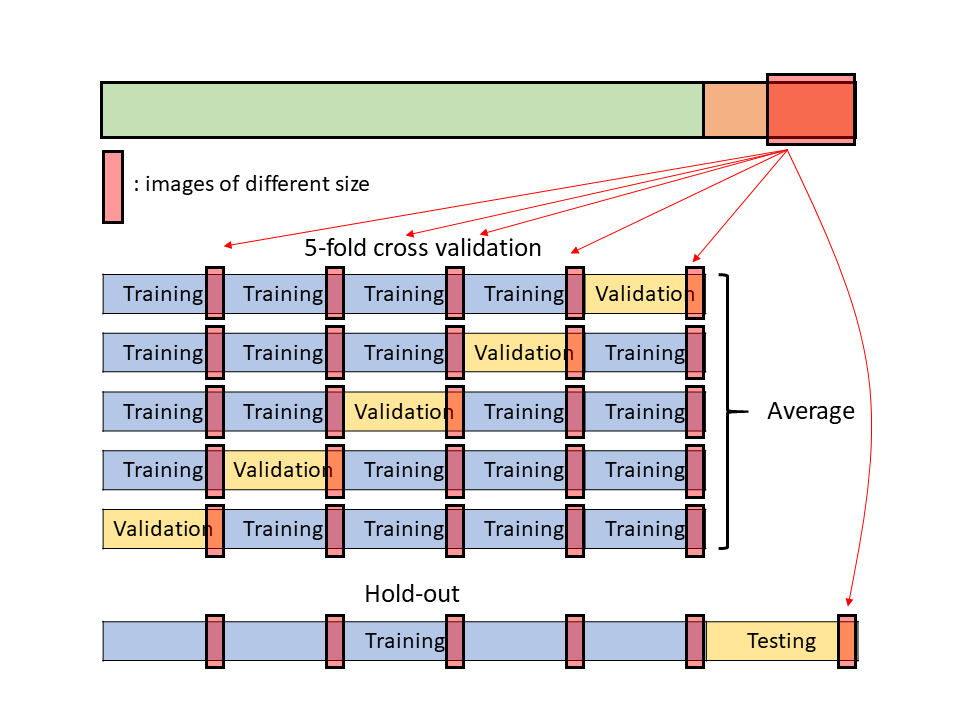

Supplement: Supplementary file 1 [file cancers-14-03334-s001.zip › cancers-1760321-supplementary/Supplemental Figures revised/e-Figure S1.TIF]

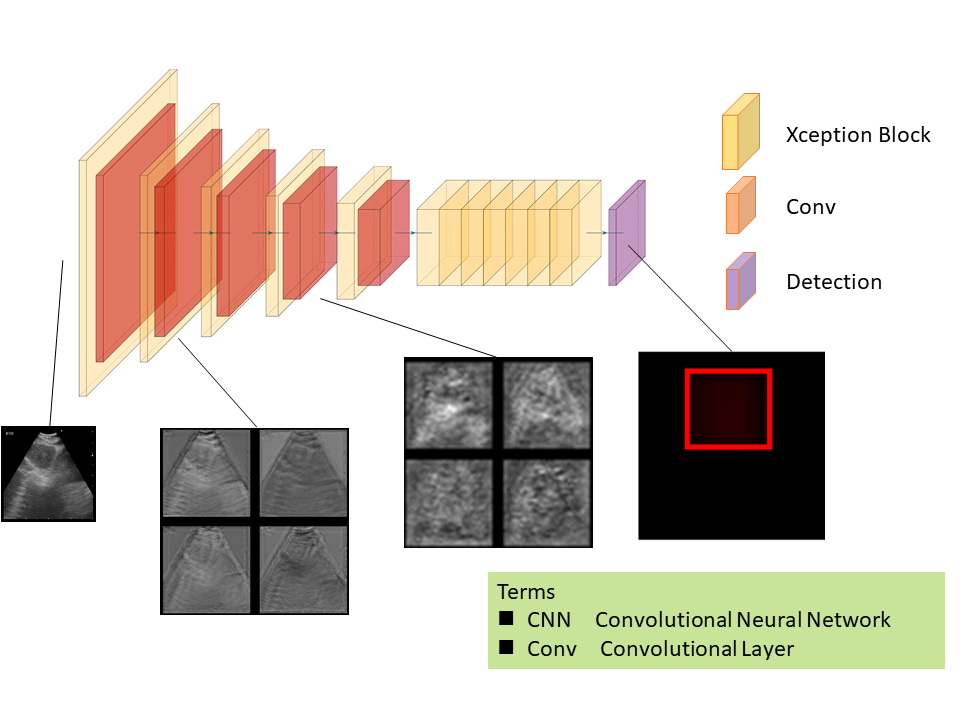

Supplement: Supplementary file 1 [file cancers-14-03334-s001.zip › cancers-1760321-supplementary/Supplemental Figures revised/e-Figure S2.TIF]

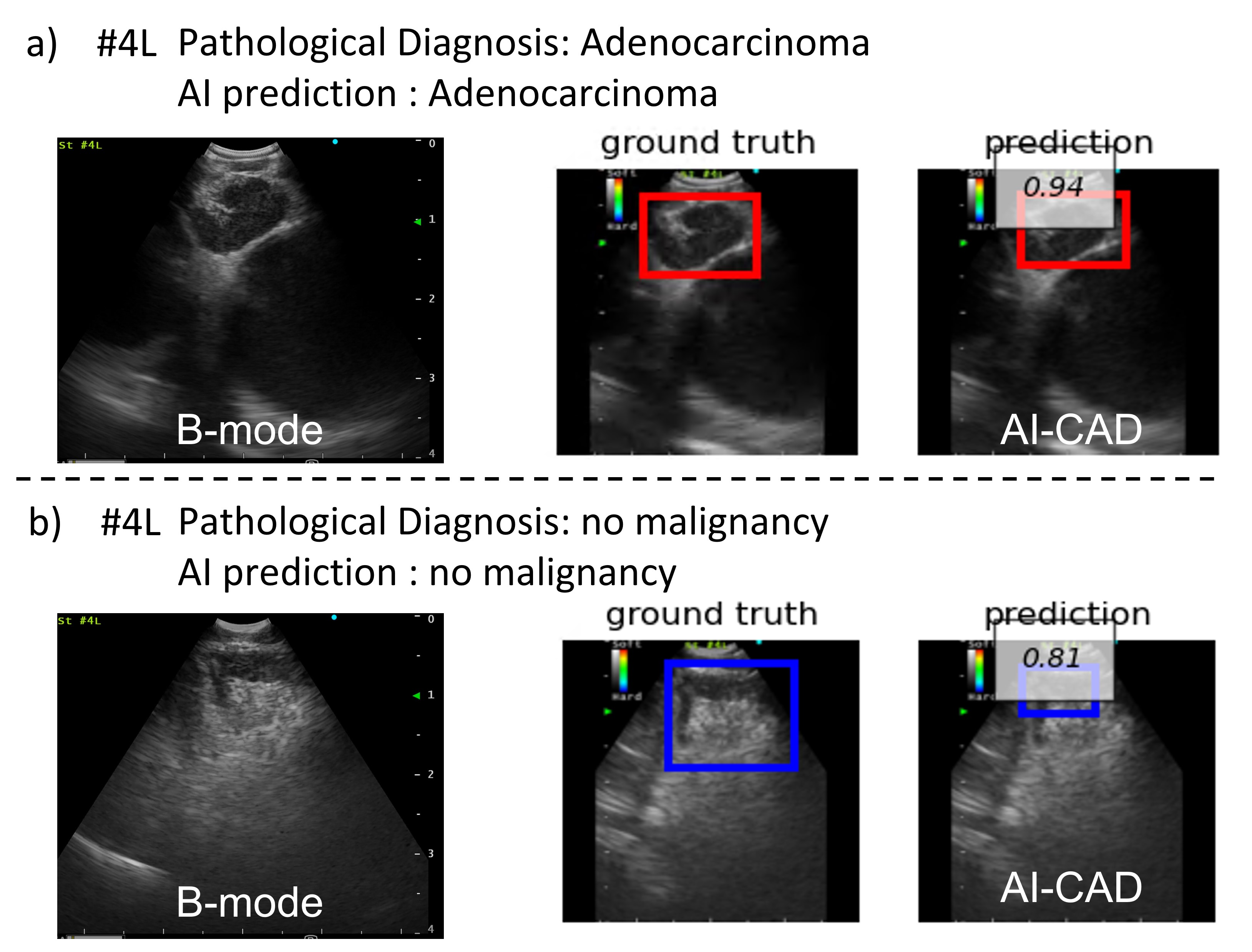

Supplement: Supplementary file 1 [file cancers-14-03334-s001.zip › cancers-1760321-supplementary/Supplemental Figures revised/e-Figure S3.tiff]
